# Dark clouds in co-creation, and their silver linings practical challenges we faced in a participatory project in a resource-constrained community in India, and how we overcame (some of) them

**DOI:** 10.1080/16549716.2017.1421342

**Published:** 2018-01-26

**Authors:** Preeti Sushama, Cristian Ghergu, Agnes Meershoek, Luc P. de Witte, Onno C. P. van Schayck, Anja Krumeich

**Affiliations:** ^a^ Department of General Practice of the Faculty of Health, Medicine and Life Sciences, School for Public Health and Primary Care (CAPHRI), Maastricht University, Maastricht, The Netherlands; ^b^ Department of Health, Ethics, and Society, School for Public Health and Primary Care (CAPHRI), Maastricht University, Maastricht, The Netherlands; ^c^ Centre for Assistive Technology and Connected Healthcare (CATCH), University of Sheffield, Sheffield, United Kingdom; ^d^ Department of Family Medicine, School for Public Health and Primary Care (CAPHRI), Maastricht University, Maastricht, The Netherlands

**Keywords:** Participatory action research, urban slums, dialogue with community, challenges, research conventions

## Abstract

**Background**: While any type of field-based research is challenging, building action-oriented, participatory research in resource-constrained settings can be even more so.

**Objective**: In this article, we aim to examine and provide insights into some of the practical challenges that were faced during the course of a participatory project based in two non-notified slums in Bangalore, India, aiming to build solutions to indoor air pollution from cooking on traditional cook stoves.

**Methods**: The article draws upon experiences of the authors as field researchers engaged in a community-based project that adopted an exploratory, iterative design to its planning and implementation, which involved community visits, semi-structured interviews, prioritization workshops, community forums, photo voice activities, *chulha*-building sessions and cooking trials.

**Results**: The main obstacles to field work were linked to fostering open, continued dialogue with the community, aimed at bridging the gap between the ‘scientific’ and the ‘local’ worlds. Language and cultural barriers led to a reliance on interpreters, which affected both the quality of the interaction as well as the relationship between the researchers and the community that was built out of that interaction. The transience in housing and location of members of the community also led to difficulties in following up on incomplete information. Furthermore, facilitating meaningful participation from the people within the context of restricted resources, differing priorities, and socio-cultural diversity was particularly challenging. These were further compounded by the constraints of time and finances brought on by the embeddedness of the project within institutional frameworks and conventional research requirements of a fixed, pre-planned and externally determined focus, timeline, activities and benchmarks for the project.

**Conclusions**: This article calls for revisiting of scientific conventions and funding prerequisites, in order to create spaces that support flexible, emergent and adaptive field-based research projects which can respond effectively to the needs and priorities of the community.

## Background

‘World and human beings do not exist apart from each other, they exist in constant interaction’ [1]. Freire’s statement is continuously reinforced in our work in Bangalore, where in collaboration with people living in two non-notified slums, Project Exhale builds participatory action towards combating indoor air pollution (IAP) from cooking on traditional stoves, called *chulhas*, in these informal communities  that are not 'notified' or recognized as slums by the Government of India, thereby exacerbating their access to basic facilities, like water, sanitation, electricity and health care [2–5]. A large part of this work involves examining contextual factors that interact with and shape people’s needs, priorities, choices and views on cooking and cooking equipment. The aim is to direct this knowledge into an iterative design process in which slum-dwellers are engaged with other stakeholders, such as researchers, industrial designers, local industrial manufacturers, to name a few, in co-creating a solution to air pollution inside houses in these informal settlements, and in addressing other issues impinging upon its adoption and sustainability [6]. In order to uncover and understand the perspectives of the communities, we rely upon unstructured observations, semi-structured interviews, focus group discussions as well as more hands-on *chulha*-building and cooking sessions in the slums.

In this project-based study conducted over 2014–2016, we draw heavily upon schools of thought and practice that adopt a democratic form of research with and for the people, rather than on them. These practices are heralded by numerous names, such as participatory action research, community-based participatory research and participatory design. They are defined in various manners: for instance, participatory action research (PAR) is described as a cyclical context-based approach to research where researchers and participants together identify a situation in need of change, devise action based on capabilities and assets [7], whereas community-based participatory research (CBPR) is viewed as ‘a collaborative approach to research that equitably involves all partners in the research process and recognizes the unique strengths that each brings’ [8]. Furthermore, these practices are increasingly viewed not as methods but as ‘*an orientation to research*’ [9]. With differing ways of defining and understanding participation under each faction, it naturally follows that there is no real map or methodology for participatory action. It can also be argued that it is rightfully so, because a rigidly defined set of goals and activities would be counterintuitive to the flexibility and emergent nature of participatory approaches [10]. It is undeniably important to rise above ‘techniques-based orthodoxy’ [11] in order to tailor participatory projects to the unique needs of the realities in which they are based. It is, however, easier said than done.

In this article, we aim to examine some practical challenges we faced in our work with two slum communities while trying to build participatory processes with them. The objective of the article is to provide insight into some of the language, cultural and contextual barriers we attempted to overcome in this project, in order to share our experiences, put forth questions and build upon the dialogue in this field with academicians, practitioners, planners and designers of participatory projects. This article is also intended to add to perspectives on the practice of participation in an under-represented section: urban non-notified slums in India.

## Dialoguing with the community

Exhale is a multidisciplinary initiative whose research arm was represented on ground by the first and second authors of this article, global health researchers from India and Romania, respectively. As field representatives of the project, we were responsible for directing the day-to-day activities, coordinating between different partners and stakeholders, and for keeping the research and design processes grounded in the context. A closer examination of this negotiation of roles of researchers, implementers, mediators, and evaluators, and their interplay with the project processes can be found in Ghergu's work (currently under review) elsewhere. While any field-based research involves ‘coping with multiple negotiations and continually dealing with ethical dilemmas’ [12], PAR and associated practices present the added challenge of the need to bridge two conflicting social worlds [7] – the ‘local’ and the ‘scientific’. Communication and language play a central role in our project, as tools for the following (it is important to note here that while a broad categorization of these processes has been used for the purpose of this article, in reality, they occur concurrently, engaging in a continuous interplay, and often building upon each other):

- The ‘us’: introducing the project and our team to the community, relaying our aims and objectives, what we are trying to do, providing a rationale or an explanation for our presence in their lives
- The ‘them’: uncovering and understanding the community’s realities, exploring and understanding their needs, priorities, as well as underlying complexities
- Bridging the gap between ‘us’ and ‘them’: overcoming distrust, building trust for an open, two-way relationship.

Communicating the purpose behind our presence in the slum and our engagement with the community had to be a careful and deliberate process which was initiated in our initial visits and conversations, and was built upon bit-by-bit through interactions with individuals, families and groups in the community over subsequent visits. Most of the initial interactions between ‘us’ and ‘them’ were mediated by a veteran social worker who had worked closely with them towards rehabilitating and bringing about development in these communities, and was known and trusted by them. His involvement was crucial to lending us credibility and overcoming the initial suspicions towards the ‘outsiders’, and we could not have initiated a relationship with the people without his active support. However, his position of authority made it nearly impossible to have an honest conversation and a dialogue as equals with the community, and the need for using third-party interpreters became increasingly evident. Indispensable to ‘co-intentional education’ where both sides are involved in ‘unveiling reality, knowing it critically and in recreating that knowledge’ and in doing research without ‘prescription’, that is, without imposing our ideas and choices upon them [1], dialoguing with the community was a complicated process that presented various challenges.

## Language barriers

The slum communities are mostly composed of migrant workers from different parts of South India, and a diverse range of dialects of Telugu, Tamil, Kannada, and in a small part, Hindi are spoken here. In order to effectively communicate with them, there is a need for translators with a working knowledge of multiple languages as well as styles. In Exhale, communication with these communities was facilitated by a host of local translators and interpreters, professional as well as amateur, conversant in English and Kannada, with little grasp over other languages. Furthermore, in a project such as ours, where establishing trust through direct, open and clear communication, the use of translators in itself was less than ideal. Various parts of the interaction between us and the community, including the transmission of our message and questions to people, and transmission of knowledge from people to us, were coloured by the perspectives, experiences and opinions of the translators, as well as their skills. While we had multiple sessions of background training with the translators in order to minimize such influences, it was difficult to remove them entirely. It was even more challenging to identify, and correct for, the degree to which assumptions made by them, whether based in logic, or founded upon their previous knowledge and experience, modified the translated response of the interviewee. For instance, in exploring views on smoke and perceptions of its impact on health, most translators had trouble talking about smoke without introducing their own interpretations into the conversation before we had a chance to hear about the interviewee’s experience and perspectives. As a result, it was challenging to separate the community’s perspectives from that of the translators at all times.

While measures such as back translation could be employed in order to ensure validity of responses, there is no way to pass communication disseminated from our side on the field through such mechanisms. Providing questionnaires and strict guidelines to translators was not preferable in our study which adopted an exploratory design, where establishing a relationship with the people was at the forefront of most initial interactions, and data was generated and collected more through a conversation between two (and, sometimes, more) groups of people, than an interview. It was also important to provide space for the interviewees to direct the dialogue and bring up their own topics of discussion, which would not have been possible with strict or rigid guidelines to be followed. The translators/interpreters needed to be given some flexibility, in order to build that relationship, to reflect, respond, and have a conversation.

We found a great resource in students of local universities and young professionals, assisting us as part-time translators. Birthed from a need for ‘pocket money’, their interest in the study quickly grew as they developed a connection to a world so well hidden within their own, that most of them were unaware of its existence mere metres from large roads and intersections that they traversed every day. More sensitive to needs of both sides and eager to listen and learn, these amateur translators were keen on connecting with the people, which formed the crux of our work with these communities.

## Incomplete information

We received incomplete answers to some of the questions we asked during the field visits. There was a multitude of reasons behind this. Often, the questions seemed odd or illogical. For instance, a lot of the women were confused when asked how they built their stoves, because to them, the act of putting together a few stones or bricks, cemented by some mud from their surroundings did not qualify as ‘building’. During sessions with foam bricks to build mock *chulhas* and discuss changes that could be made, most attempts to try top-lit designs were met with resistance, because to the participants, habituated to using bottom-lit *chulhas*, it appeared to be highly illogical. Sometimes, this sense of oddness was also shared by the interpreters who did not always fully understand the point of pursuing lines of investigation such as perceived effects of smoke on the people, since it was ‘obviously bad’. In other instances, some women did not elaborate much on questions about cooking processes or questions dealing with household responsibilities, because they were sure that as an Indian woman, the researcher already knew the answer and did not need much explanation. We observed that most challenges arose when people were asked to vocalize the process of cooking. The act of cooking is so habituated that it was difficult to verbalize their actions, or explicitly point something out. We had to rely on observations, and make indirect inferences, which were in turn situated in our knowledge and understanding of cooking and cooking practices, and bound by our definitions of what is relevant and what is not, and hence embodied ‘a larger element of risk and uncertainty than with more formal methods’ [11].

Some questions and responses were lost in translation. Some participants were shy, or simply unwilling to answer, while a lot of them were distracted by children or neighbours. Finally, sometimes, there were simply no answers; whether this was because the questions did not make sense to them, or because it dealt with issues upon which they did not want to have a conversation with us, we can only speculate. Following up on interactions with incomplete or confusing replies was difficult, since the challenges involved in picking up threads of previous conversations were compounded by the transient nature of the community, especially in their location and the hours of their availability.

## Incentives for participation

In 2014, indoor air pollution was recognized by the WHO as the world’s single largest environmental risk to health, accounting for 4.3 million deaths worldwide, and linked to a wide range of diseases such as chronic pulmonary obstructive disorders, lung cancer, and cardiovascular diseases, among other adverse health impacts [12]. A major contributor to the issue was found to be the smoke released from cooking on open fires and traditional stoves, a practice that, in 2014, was being used by 700–800 million Indians. Non-notified urban slums, with constrained resources and little or no access to subsidies on cleaner fuels, were seen to be particularly reliant on firewood and other biomass fuels for cooking and thereby are at a disproportionate risk to its dangers [6,13]. Project Exhale was born from and driven by this epidemiological evidence of risks that indoor air pollution poses to health and living environment in slums. The motivation of its members is rooted in these considerations as well as their conviction that people’s participation in the design and implementation of any solution to the issue is indispensable to the process. But what does this externally conceptualized project mean to the people? Why would they be motivated to participate in an initiative centred around an issue that for the community was low in priority? How do we move beyond ‘window-dressed’ participation [14]?

We tried to identify areas that would lend meaning to their involvement in the project, by drawing upon literature and experiences of other community-based *chulha* projects [15–20,21] as well as our interaction with the people in the slums. While some were aimed at directly incentivizing participation in workshops, such as providing raw materials for cooking during observation sessions, and the cooks taking home the cooked items, others were less tangible. For instance, the community forums and the photo voice sessions, wherein the communities were provided with digital cameras and asked to capture any part of their day that they viewed as relevant to their cooking practices, were tools for giving voice to their daily lives and priorities. It helped to uncover aspects that held meaning to them but had been unexplored by us. For instance, it prompted us to shift focus from smoke from cooking as a health risk to the impact the smoke and the soot have on the walls of their homes as well as the time it adds to the cleaning of pots and pans. Furthermore, as a token of appreciation for their time and inputs, families participating in cooking sessions were given gifts of sentimental value, such as framed family photos. As a means to facilitate the vocalization of the cooking process, as a way to gather information on varying cooking practices and cultures, community cooking sessions were arranged, wherein two or more women cooked on different prototypes at the same time. Observations were made while they were cooking, and post-cooking interviews were held with the cooks to gain feedback on the use of the *chulha*. Neighbours were also encouraged to provide their reactions as spectators, for instance, on how they expected the prototypes to perform, and how they perceived the comfort of cooking on each *chulha*.

Apart from these, and a much more important ‘return’ for the time and energy invested by the community, we tried to ensure that the project was defined and directed by the priorities of the people, be it financial, infrastructural, social or personal. Complex and varied as they were, balances between conflicting needs and beliefs had to be found over time through continuous communication and some compromises. A further in-depth, rigorous study of these dynamics is recommended.

## Demands on scarce resources

Participation is increasingly viewed as more than just a tool for project implementation, but rather as a philosophical approach that benefits all the involved parties and that ‘creates the possibility of the exercise of citizenship’ [22, p.xi], a sentiment echoed by our local liaison in saying that any initiative in these settings ‘*…should be a participatory programme, from planning to implementation. Otherwise we are doling (out) and people are taking. Spoon-feeding should not happen*.’ However, it is important to recognize that this representation from the community draws upon their time, money, and energy – all limited, precious resources in this setting.

In a community characterized by transience, uncertainty and insecurity, time is money [23]. The morning routine in a slum household is packed and tailored to best navigate the chaos emerging from an attempt to juggle multiple roles and responsibilities within a limited time frame. Women and older girls are in charge of cooking food for the family, usually enough to last for the day, feeding the younger children, helping others get ready for work or school, and then leaving for work by 8.30 am themselves. To make space for ‘participation’ in this well-oiled machinery risks throwing the whole system in disarray, and linking the resulting confusion and frustration to project activities. We found from another participatory chulha initiative based in rural Karnataka, that they tailored the amount of time the women had to put in to the project to the minimum acceptable, so as to avoid any ‘unnecessary annoyance’ to them. The engineer who pioneered this social design project emphasized upon the need to be ‘on the same side of the line’ as the slum-dwellers in order to build co-ownership, that it was important to invite them to participate in the activities occurring outside of the slums, for instance, trials with prototypes at the offices of the designers. But to gain ideal levels of participation raises questions of distance and time involved in travel (particularly in a large city like Bangalore), as well as loss of wages for work missed and the associated need for compensation. For the aforementioned project, distance was not much of an issue, as their work mostly occurred in small villages, and while they dealt with the dilemmas of financial loss by tying up their measures to counter indoor air pollution with employment generation initiatives within a forest conservation drive, the resources and constraints of the project within the urban context did not present such opportunities to us.

## Conflicts/tensions at work

Action research is a field ‘where “subjects” are viewed as partners in the research process – to dupe them in any way would be to undermine the very processes one wants to examine’ [11], and hence, it was essential for us to be open and honest with the communities at all times. However, we often found it challenging to achieve full disclosure in the face of uncovering and understanding people’s realities without biasing them or affecting their responses. As relatively young researchers in a fairly new environment, dilemmas such as what to say when one is asked questions about the study, what benefits the participants would gain from the research, and if and how we could (or would) help them ‘often had (sic) to be resolved “situationally” and spontaneously, without the luxury of being able to consult with a more experienced colleague’ [11]. Punch’s advice to researchers in these situations is to ‘enter the field with a nebulous explanation of (your) purpose… it is not “ethically necessary or methodologically sound, to make known… particular areas of interest”’ [11]. However, in order to avoid conflicts later due to ambiguity and differences in expectations and outcomes, ‘clarity through specificity’ [14] is important. Where, then, does the line lie?

Tensions at work also arose due to divergences in focuses and priorities. Although ideally, in PAR, researchers and participants work together to ‘identify a situation in need of change’, project Exhale and its activities were motivated by epidemiological evidence, and was not based in the people’s expressed needs. In a community struggling with access to basic necessities, attentions and energies were preoccupied with emergencies of poverty, lack of water and electricity, and monsoon-related disease outbreaks. ‘A bit of smoke’ that is viewed as a natural, albeit annoying, part of cooking, rather than a veritable risk to health [6] did not take precedence under these circumstances, and our focus upon it caused some frustrations, as echoed by one man from the slum, during a group interaction: ‘*You have been here, you have been working here for one year. People are dying, because of lack of money – that is the immediate problem, and it needs an immediate solution*’.

## Conclusions

In 2014, during the time when the project was conceived, there was a lot of attention from the scientific community on indoor air pollution as a global health risk, and the growing interest in studies and initiatives on the issue resulted in varied funding opportunities in the area. Embedding this work within PhD programs made it feasible to initiate action on health and development in slums; it was our proverbial foot-in-the-door. At the same time, however, it constrained the project within a target- and time-bound plan which is counterintuitive to the open-ended, iterative and emergent nature of an ideal participatory process, in addition to constricting the comprehensive nature of the intervention that such a setting calls for. It had an impact upon how research was done, how data were collected, the data itself, and as a result, the action taken. Staying true to this process and dealing with the dilemmas and conflicts raised by the complex setting, while under pressure to answer to, and report step-by-step on the progress made and ‘goals’ met, to funders and other parties who are invested in this project, while racing against the defined timeline to meet the requirements of a three-year PhD study, is a constant struggle and makes this work complex and challenging. However, it is our unwavering belief that there is no better alternative to participatory, action-based research, since conventional approaches to research and development entail the same limitations and more.

In an ideal setting, the focus of the project would not have been primarily restricted to indoor air pollution, but instead would have been drawn from the community itself and would have included the network of interacting issues that form preoccupations for the people, both in the short term, such as unavailability of drinking water and electricity, and in the long term, for instance, employment and livelihood issues. It would have involved collaboration with a wider network of actors, specializing in tackling varied issues, and the work would have been carried out by researchers with greater familiarity with the local languages, cultures and contexts. Most importantly, the interventions as well as the timeline of the action would not have been pre-decided, but rather would have been built upon the preliminary knowledge of needs and priorities that was gathered in the early stages of the project and would have been designed collaboratively with the community. For this to occur effectively, what is needed is the recognition of the numerous complexities involved in the practical applications of PAR, and a revisiting of conventions of pre-designed plans, goals and standardized time limitations on participatory projects, in order to make way for new research processes that give researchers and other practitioners the kind of flexibility that is necessary to meet the complexities of the context, to build collaborative action that focuses upon the interacting network of issues that plague slum-dwellers, to work with the communities and bring about change that truly responds to their needs and priorities.

This challenge is not a new one; Stephen Corey wrote in 1949 that in ‘a program of action research, it is impossible to know definitely in advance the exact nature of the inquiry that will develop. If initial designs, important as they are for action research, are treated with too much respect, the investigators may not be sufficiently sensitive to their developing irrelevance to the ongoing situation’ [24]. More and more contemporary authors and practitioners are dealing with similar practicalities, facing ‘the dilemma of how to present participatory research in a way that is recognisable to august bodies without affecting the quality of our research’ [25], and are pushing for changes in how research is being done. They emphasize that methodological processes are important, but new conventions on categories and practices in field- and action-based research as well as standards used to evaluate their value and validity at the time of funding applications are also essential [25]. A rigid emphasis upon scientific methods cannot come at the cost of the needs of the community and the context in which the action research is being done.

## Paper context

This article examines practical challenges faced in building participatory processes in vulnerable, neglected populations: urban non-notified slums in Bangalore, India. While the main difficulties arose from the dynamic complexities of the resource-poor setting, these were reinforced by traditional scientific conventions that bound the study in terms of time, money and scope. To strengthen and support the ability of action-oriented research to respond to and build upon the needs and priorities of the people, these conventions need to be revisited and re-examined.
